# A patient with recurrent syncope—it does matter how slow and long you go

**DOI:** 10.1007/s12471-023-01778-z

**Published:** 2023-05-03

**Authors:** Eva Roseboom, Alexander H. Maass, Jozine M. ter Maaten

**Affiliations:** Department of Cardiology, University Medical Centre Groningen, University of Groningen, Groningen, The Netherlands

An 81-year-old male presented to the emergency department with recurrent syncope. Two weeks prior, he had undergone successful elective cardioversion for atrial flutter, and his medication was switched from bisoprolol 2.5 mg once daily to sotalol 80 mg three times a day. During transport to the hospital, he was resuscitated and defibrillated. Upon presentation to the emergency department, the patient experienced no cardiac symptoms and was hemodynamically stable. Physical examination revealed no abnormalities except for a decreased heart rate of 40 bpm (Fig. 1a). Laboratory investigation showed borderline normal values of magnesium (0.71 mmol/l: reference values: 0.70–1.00) and potassium (3.5 mmol/l; reference values: 3.5–5.0) and mildly reduced kidney function (serum creatinine: 114 μmol/l; reference values: 50–110). While at the emergency department, he lost consciousness. The electrocardiogram (ECG) recorded at that time is shown in Fig. 1b.

**Fig. 1:**
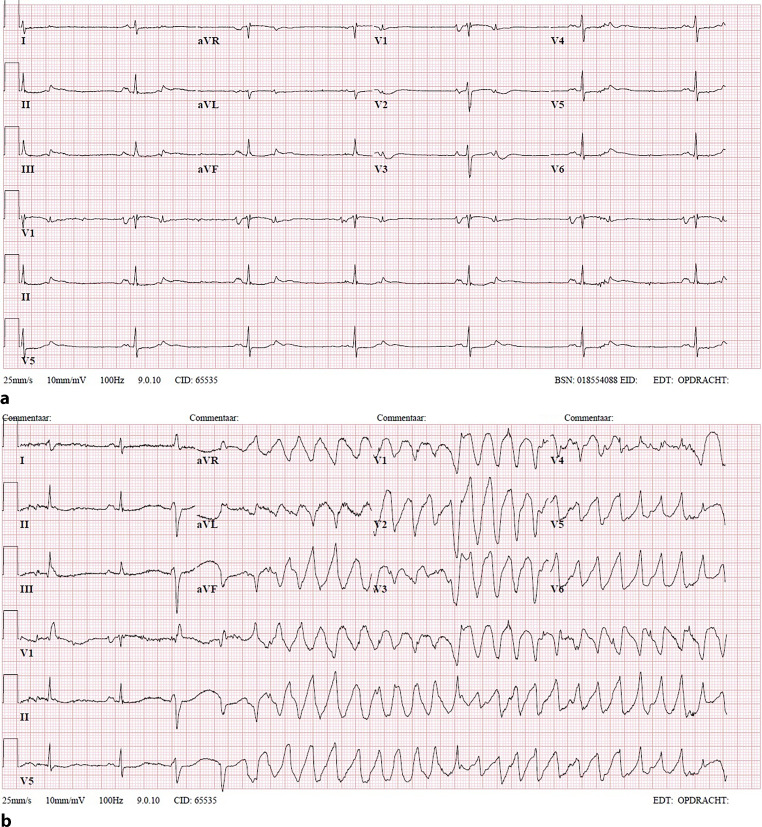
Electrocardiograms (**a**) at presentation at the emergency department and (**b**) during syncope

How would you explain the T wave abnormalities in the first ECG (Fig. 1a), and what is the mechanism of the tachycardia visible in the second ECG (Fig. 1b)?

## Answer

You will find the answer elsewhere in this issue.

